# The Threatened Strike of Asylum Nurses

**Published:** 1913-10-11

**Authors:** 


### THE NURSING DEPARTMENT.

### The Threatened Strike of Asylum Nurses.

### A BLOW TO THEIR PRESTIGE AND POSITION IN THE NURSING WORLD.

For many years there was a feeling among hospital-trained nurses that the asylum-trained nurse is inferior in status. The asylum-trained nurse has not been accepted as an equal, but has been rather looked down upon as imperfectly educated, uncouth in manners, and insufficiently trained. This feeling has of late years been dying away, but it is to be feared that it will be resuscitated in full intensity by the threatened strike of the nurses in the West Riding. Forty years ago the hospital-trained nurse and the asylum-trained nurse were much on a par. Both were drawn from a lower social stratum than nurses come from nowadays; both were devoid of any special training. A time came, however, a time closely coincident with the adoption of Listerism, when mere clinical experience without systematic training was found insufficient to meet the demands made upon the nurse by the surgeon, and at the same time the new spirit of enterprise that awoke in women of gentle birth impelled many, whether compelled by necessity or urged by desire for useful occupation, to look for an outlet for their energies in other directions than those of teaching young children and becoming companions to old ladies. Thenceforward the wards of hospitals were thronged with young women, the daughters mainly of professional men, gentlewomen of the same social status as the medical students who were being trained alongside of them, and whose training, if it extended over a wider field, was far less severe. These women entered upon their career equipped with the general education that was then given, in what are now called secondary schools, to the girls of the upper middle class, and this education was supplemented by a professional education, carefully given by means of lectures, demonstrations, examinations, and so forth, in the hospital wards and class-rooms.

It was some twenty years later before this movement invaded the world of asylums. It was not until 1893 that the Medico-Psychological Association started its system of training and examination, and, although before that sporadic attempts had been made here and there to give systematic training in a few asylums, the great bulk of asylum nurses were without any systematic instruction, and gained efficiency solely by experience in the wards. This has been remedied, and the course of instruction that is given to asylum nurses is now at least as full and thorough in its own department as that given to hospital nurses, and it has the merit that the qualifying examinations are held by an independent body, and are uniform throughout the kingdom. In a small or a slackly administered hospital it is possible for a nurse to obtain a certificate of competency on inadequate training and knowledge, but the certificate of the Medico-Psychological Association ensures that its holder is, as far as knowledge and training go, a competent mental nurse.

The other movement—that for raising the status of nurses and drawing them from a higher social stratum—has made but little way in the asylum world. There are very few asylums in which ladies are trained as nurses and there is a very small number of gentlemen who are competent to act as nurses to the insane; but both men and women nurses in asylums are, as a rule, persons who would feel more at home in the servants' hall than in the dining room, who could not sit comfortably at meals with gentlefolks, and would not be at ease with dinner napkins and finger bowls. This is much to be deplored. The nurse of a mental case should be more of a companion and associate of the patient than the nurse of a surgical or medical case. More is needed. The nurse must before everything obtain the trust and confidence of the patient, and be on terms of fellowship and equality at least, and if of superiority so much the better. However skilled a nurse may be in technical requirements, he or she is of little use in many mental cases without a mental and social equipment at least equal to that of the patient. To place a man of education and refinement in the daily and hourly companionship of a kindly and technically skilled, but uneducated and mannerless boor would be galling to the patient, and could not promote his peace of mind or conduce to his recovery.

The difference between the medical and surgical nurse on the one hand and the mental nurse on the other is brought out and made conspicuous by the threatened strike among the nurses of the asylums in the West Riding. Such a movement would be impossible—would be scarcely conceivable—in a hospital or a group of hospitals. Nurses, like other people, have the right to demand and secure adequate remuneration for their time, labour, and skill; but that nurses should seek to secure these ends by means of a strike—by leaving sick, helpless, and suffering patients unattended—is utterly unjustifiable and abominable. It would be unjustifiable and abominable in an ordinary hospital for the sick, but when to all the horrors of neglect of the sick are added neglect of the suicidal and violent the proposal is one that it is difficult to speak of with restraint or moderation. Experience has shown that the success of strikes is largely dependent on the sympathy that the strikers can secure from the public at large. In such a strike as is threatened in the West Riding the strikers are secure of the most whole-hearted condemnation from the outside public. The mere threat is enough to revivify and confirm the general opinion that was in course of extinction—that mental nurses are an inferior lot. Such an opinion has been prevalent. We have always believed that it was mistaken; but if this strike should take place mental nursing will be branded with a stigma from which it will scarcely ever recover.

